# Identification of LMO2 as a new marker for acinic cell carcinoma of salivary gland

**DOI:** 10.1186/s13000-022-01192-w

**Published:** 2022-01-30

**Authors:** Dong Sheng, Yan Zhang, Tian Xue, Xiao-Yan Zhou, Xiao-Qiu Li

**Affiliations:** 1 Department of Pathology, Fudan University Shanghai Cancer Center, 270 Dong-An Road, Shanghai, 200032 China; 2 Department of Oncology, Shanghai Medical College, Fudan University, Shanghai, China

**Keywords:** Acinic cell carcinoma, Secretory carcinoma, Salivary gland, LMO2

## Abstract

**Background:**

The distinction between acinic cell carcinoma (ACC) and secretory carcinoma (SC) of the salivary gland is hampered by the lack of specific diagnostic markers. It is known the cytoplasm of glandular cells in the salivary gland immunohistochemically expresses LIM Domain Only 2 (LMO2). Herein, we aim to evaluate the expression status of LMO2 in a large cohort of tumors of the salivary gland, with an emphasis on its significance in the distinction of ACC and SC.

**Methods:**

Immunohistochemical stains were performed to evaluate the expression of LMO2 in normal tissues and tumors of salivary gland.

**Results:**

LMO2 was expressed in normal serous acinar cells of the salivary gland. We also found the cytoplasmic immunostaining of LMO2 was specific and sensitive for the recognition of ACCs including those with morphological overlaps with SCs, whereas the cytoplasmic expression of LMO2 was not detected in SCs.

**Conclusions:**

LMO2 is useful for the recognition of ACC and is of potential value in distinguishing ACC from SC.

**Supplementary Information:**

The online version contains supplementary material available at 10.1186/s13000-022-01192-w.

## Introcuction

Acinic cell carcinoma (ACC) of the salivary gland is generally considered a low-grade tumor with high recurrence rates [1]. While conventional ACCs feature serous acinar cell differentiation and rich cytoplasmic zymogen granules, the proportion of the neoplastic serous acinar cells varies considerably from case by case, and some cases may comprise additional neoplastic intercalated duct-resembled cells, vacuolated cells or clear cells. In addition, the morphologic diagnosis of high-grade transformed ACCs that lack pathognomonic serous acinar cells or zymogen granules-poor ACCs (prominent intercalated duct-like pattern) can be difficult [2, 3]. Therefore, the distinction between ACCs and other tumors of the salivary gland, especially secretory carcinomas (SC), may pose a significant challenge due to their histomorphologic similarities. It has been characterized that SC possesses the *ETS variant 6* (*ETV6*) gene rearrangement, which can be demonstrated by a fluorescence in-situ hybridization (FISH) [4], whereas specific diagnostic markers for ACCs and immunohistochemical markers for distinguishing these two types of salivary gland tumors need to be further explored.

LIM Domain Only 2 (LMO2) is an evolutionarily conserved protein involved in family of the transcription factor necessary for hematopoiesis and angiogenesis [5]. High level of LMO2 has been detected in various organs or tissues including normal glandular cells of the salivary gland according to the publicly available data set of the Human Protein Atlas (http://www.proteinatlas.org). Moreover, LMO2 has been reported including normal glandular hematolymphoid tumours includinga subset of germinal-center-derived B-cell lymphomas and the T lymphoblastic leukemia/lymphoma [6, 7]. Nevertheless, the applicability of LMO2 in the diagnosis of tumors of the salivary gland, has never been assessed.

In the current study, we validate the diagnostic usefulness of LMO2 by assessing the expression status of LMO2 in the normal tissues and tumors of the salivary gland, including ACCs and SCs.

## Methods

### Patient selection

A total of 176 eligible cases, including ACC (*n* = 30), SC (*n* = 11), mucoepidermoid carcinoma (*n* = 22), adenoid cystic carcinoma (*n* = 26), polymorphous adenocarcinoma (*n* = 13), salivary duct carcinoma (*n* = 15), basal cell adenocarcinoma (*n* = 1), myoepithelial carcinoma (*n* = 2), epithelial-myoepithelial carcinoma (*n* = 2), pleomorphic adenoma (*n* = 15), basal cell adenoma (*n* = 15), myoepithelioma (*n* = 1), warthin tumor (*n* = 15) and oncocytoma (*n* = 8), were retrospectively reviewed and studied. All tumors were excised specimens and were diagnosed between 2008 to 2018. Diagnostic confirmation was obtained through a review by three independent pathologists (D.S., Y.Z., and X.-Q.L.) according to the criteria of the 2017 revised World Health Organization classification. In addition, ACCs were classified as conventional low-grade ones or high-grade transformed tumors in line with observed pleomorphism, necrosis or mitotic counts [2, 8]. ACCs with paucity of zymogen granules (prominent intercalated duct-like variant) were identified as tumors with a low proportion of serous acinar cells but a high proportion of intercalated duct-like cells, and without an *ETV6* gene rearrangement [9].

### Fluorescence in situ hybridization

In the current study, FISH was carried out on the formalin-fixed and paraffin-embedded (FFPE) specimens of ACCs and SCs. Detection of *ETV6* gene rearrangement was performed with a Vysis LSI *ETV6* Dual Color Break Apart Rearrangement Probe (Abbott Molecular, Des Plains, IL) according to the protocols specified by the manufacturer. A total of 50 interphase nuclei were counted for each hybridization test. The presence of *ETV6* gene breaks was defined as follows: the presence of > 20% split signals observed in the tumor cells.

### Immunohistochemistry

Antibodies including LMO2 (1A9–1, Roche/Ventana, Tucson, AZ, USA), DOG1 (SP31, Maxim, Fuzhou, China), and S-100 (Dako, Santa Clara, CA, USA) were applied according to the manufacturer’s instructions [4, 10–12]. FFPE specimens of all the tumors as well as normal salivary gland tissues were immunostained. According to the prior studies, a percentage of > 30% of tumor cells with cytoplasmic staining of LMO2 was considered to be positive expression [7]. Whereas nuclear or membranous staining was regarded as nonspecific. The immunostaining of DOG1 and S-100 was evaluated as previously described [12]. All the IHC results were confirmed by three independent pathologists (D.S., Y.Z., and X.-Q.L.).

### Statistical analysis

Fisher’s exact test was used with GraphPad Prism 9 and results were considered to be statistically significant at a *P-value* < 0.05.

## Results

### The clinicopathologic features of patient cohort

The clinicopathologic characteristics of the 30 cases with ACC of salivary gland were summarized in Table 1. A considerable number of cases were classic low-grade ones, whereas high-grade transformation was only observed in one case. And 4 cases with zymogen-poor tumors were identified. The clinicopathologic details of the 11 SC cases were shown in Table 2. The immunostaining of the ACC marker DOG1 showed positive results in 24 ACCs, and was weakly positive in 5 additional ACCs and focally positive in 3 SCs, and the SC marker S-100 was expressed in 10 SCs and weakly positive in 4 ACCs, possibly suggesting that both antibodies are not very specific for the diagnosis. *ETV6* gene rearrangement was detected in all SC cases but none of ACC cases.

**Table 1:** Clinical and pathological features of ACCs of salivary gland

**Case ID**	**Sex**	**Age (yr)**	**Localization**	**Size (cm)**	**Subtype**	**LMO2**	**DOG1**	**S-100**	***ETV6 rearrangement***
1	F	34	parotid gland	0.8	Classic	+, C	+	-	-
2	F	58	parotid gland	2.2	Classic	+, C	+, W	-	-
3	F	42	parotid gland	1.5	Classic	+, C	+, W	-	-
4	F	51	parotid gland	0.9	Classic	+, C	+, W	-	-
5	F	19	parotid gland	2.6	Classic	+, C	+	-	-
6	F	24	parotid gland	1.5	Classic	+, C	+	-	-
7	M	58	parotid gland	2	Classic	+, C	+	-	NA
8	F	58	parotid gland	1.7	Classic	+, C	+, W	-	-
9	F	68	parotid gland	3.5	Classic	+, C	+, W	-	-
10	F	65	parotid gland	2.5	Poor zymogen granules	+, C	+	+, W	-
11	F	61	parotid gland	3	Poor zymogen granules	+, C	+	+, W	-
12	M	55	parotid gland	1.6	Classic	+, C	+	+, W	-
13	F	54	parotid gland	2.8	Classic	+, C	+	-	NA
14	M	67	parotid gland	1.7	Classic	+, C	-	-	-
15	M	65	parotid gland	4	High-grade transformation	+, C	+	-	-
16	M	50	parotid gland	2.5	Classic	+, C	+	-	-
17	F	46	parotid gland	3.2	Classic	+, C	+	-	-
18	F	57	parotid gland	5	Classic	+, C	+	-	-
19	F	23	parotid gland	3	Classic	+, C	+	-	-
20	F	31	parotid gland	2.5	Classic	+, C	+	-	-
21	M	73	parotid gland	4.5	Poor zymogen granules	+, C	+	-	-
22	F	44	parotid gland	2.5	Classic	+, C	+	-	-
23	M	67	parotid gland	3.5	Classic	+, C	+	-	-
24	F	64	parotid gland	4	Classic	+, C	+	-	-
25	F	79	parotid gland	3	Poor zymogen granules	+, C	+	+, W	-
26	F	60	parotid gland	1.2	Classic	+, C	+	-	-
27	F	51	parotid gland	1.5	Classic	+, C	+	-	-
28	F	28	parotid gland	2.4	Classic	+, C	+	-	-
29	F	29	parotid gland	1.5	Classic	+, C	+	-	-
30	M	15	submandibular gland	2.5	Classic	+, C	+	-	-

*M* male, *F* female, *C* cytoplasmic, *W* weak, *NA* not available

**Table 2:** Clinical and pathological features of SCs of salivary gland

**Case ID**	**Sex**	**Age (yr)**	**Localization**	**Size (cm)**	**LMO2**	**DOG1**	**S-100**	***ETV6 rearrangement***
1	F	62	submandibular gland	3	+, N	-	+	+
2	M	27	parotid gland	2	+, N	+, F	+	+
3	F	34	parotid gland	2.2	+, N	-	+	+
4	M	17	parotid gland	1.5	-	+, F	+	+
5	M	40	parotid gland	1.5	-	-	+	+
6	M	61	parotid gland	3	+, N	-	+	+
7	M	42	parotid gland	3	+, N	-	+	+
8	M	40	parotid gland	1.8	-	-	+	+
9	M	24	submandibular gland	1.5	-	-	+	+
10	M	27	submandibular gland	2	-	+, F	+	+
11	M	57	parotid gland	2	-	-	-	+

*M* male, *F* female, *N* nuclear, *F* focal

### The expression of LMO2 in normal salivary gland tissues

**Fig. 1:**
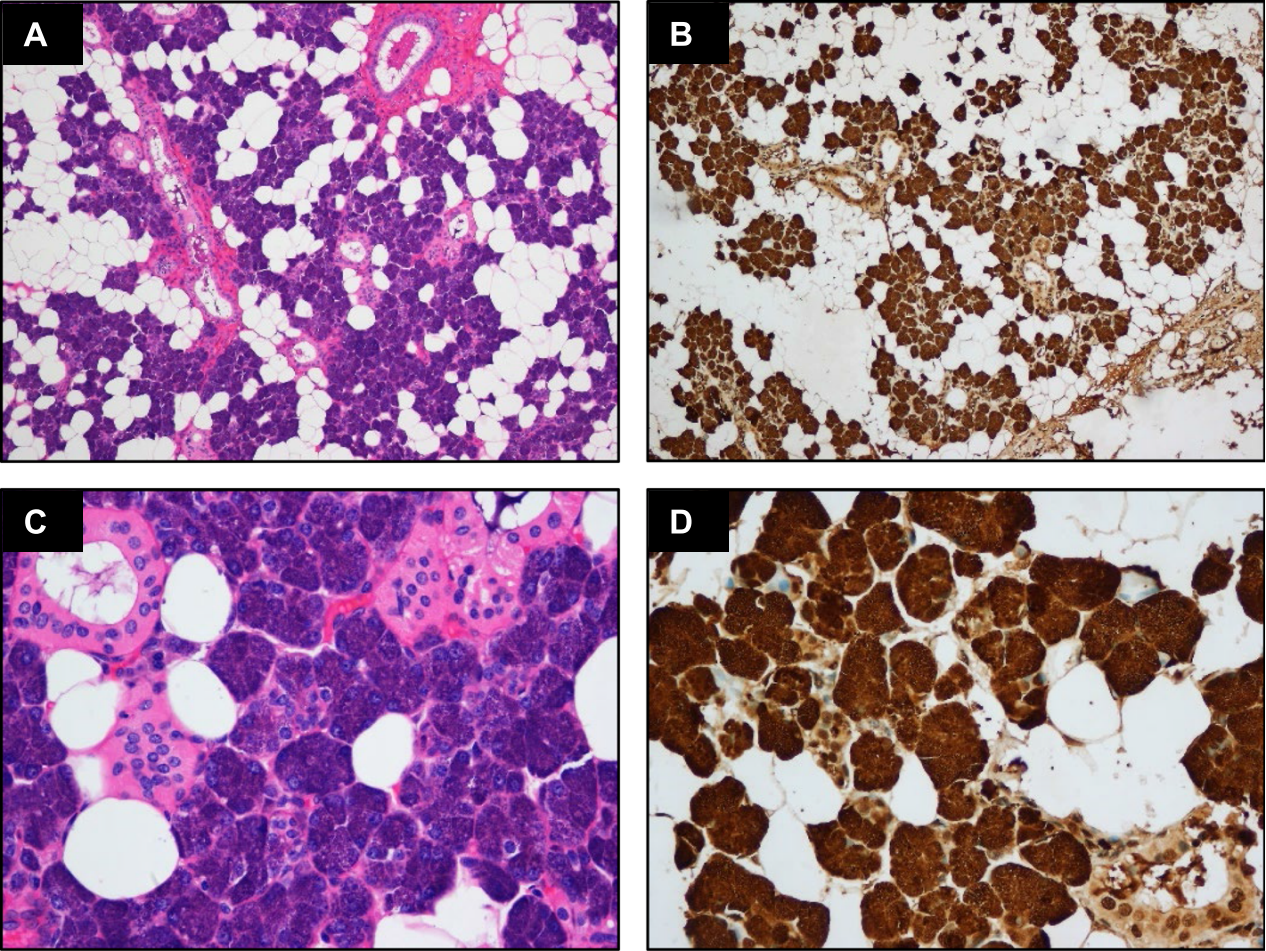
(**A**) Histology of normal salivary gland tissue. (**B**) The normal serous acinar cells of the salivary gland shows strong and diffuse cytoplasmic staining of LMO2. (**C**-**D**) Higher power view

To assess whether LMO2 was expressed in normal salivary gland, IHC detect on using in FFPE samples of 10 normal salivary glands was performed. All the specimens showed consistent and intensive cytoplasmic staining of LMO2 in serous acinar cells (Fig. 1), whereas other cell types showed negative or sometimes weak positive results (Fig. 1).

### The expression of LMO2 protein in tumors of the salivary gland

**Fig. 2:**
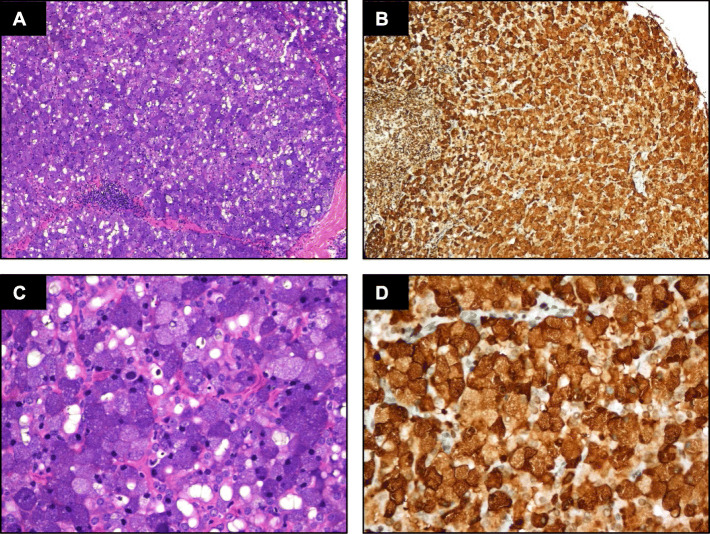
(**A**) Histomorphology of a classic ACC. (**B**) The neoplastic cells express LMO2 in high levels. (**C**-**D**) Higher power view

**Fig. 3:**
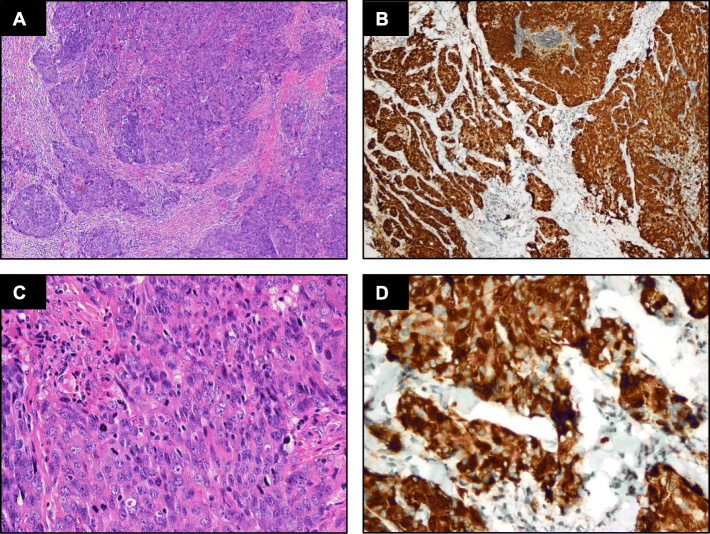
(**A**) Histomorphology of a high-grade transformed ACC. (**B**) Intensive cytoplasmic LMO2 staining is seen in neoplastic cells. (**C**-**D**) Higher power view

**Fig. 4:**
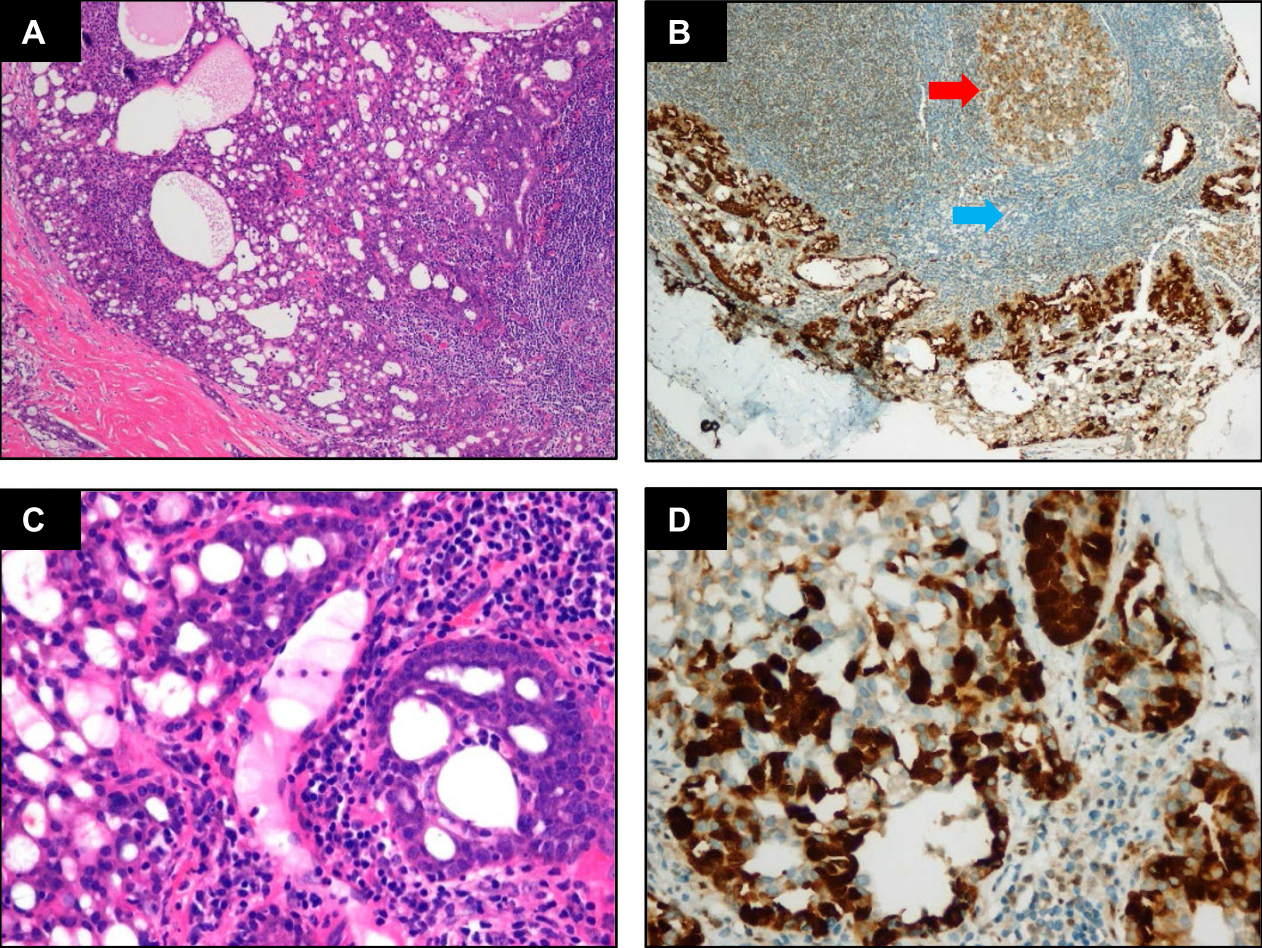
(**A**) Histomorphology of a ACC with  paucity of zymogen granules. (**B**) Strong cytoplasmic LMO2 staining is seen in the neoplastic cells. The nuclear expression of LMO2 in the germinal center B cells may serve as internal positive controls (arrow). (**C**-**D**) Higher power view

**Fig. 5:**
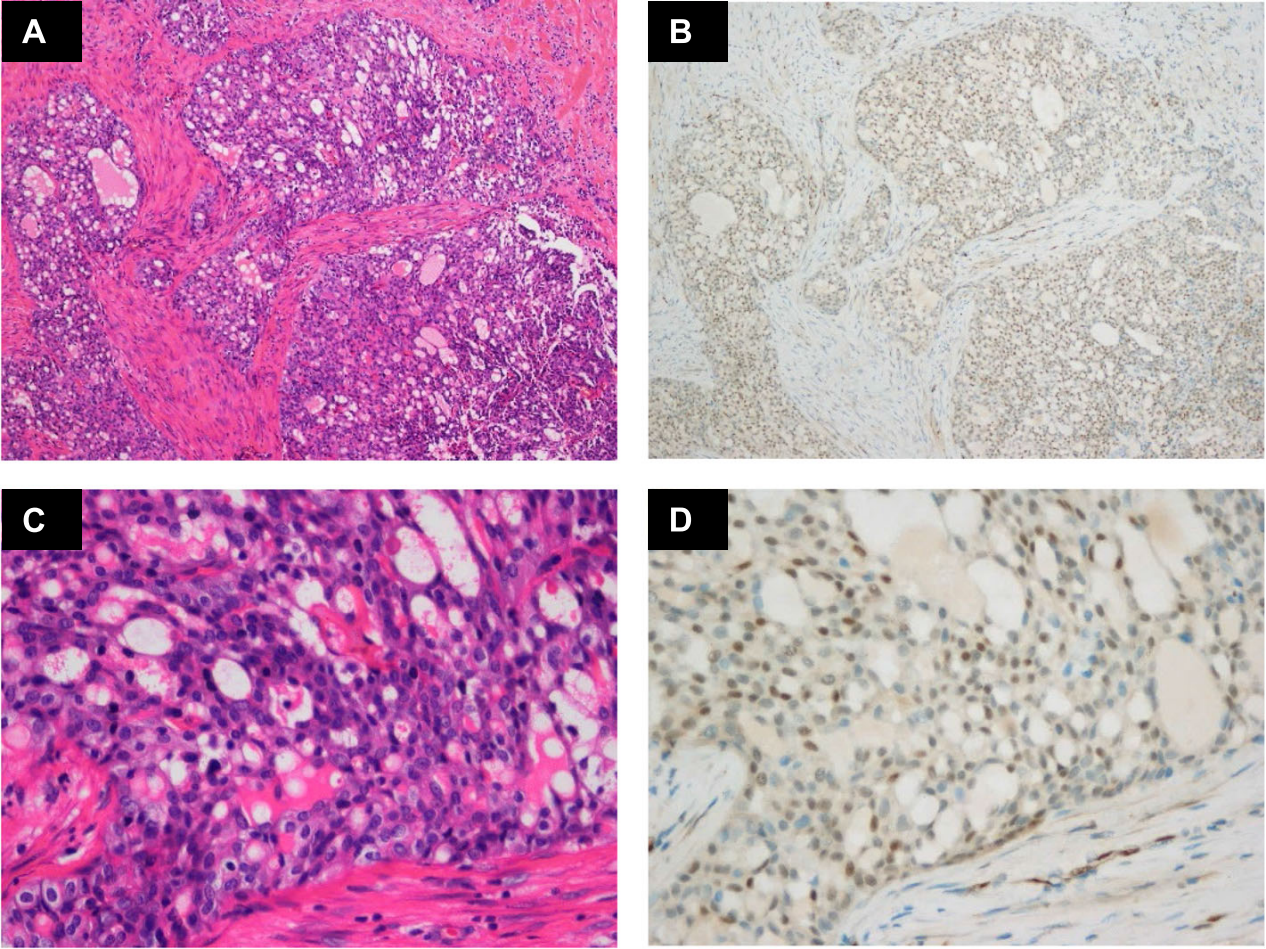
(**A**) Histomorphology of a SC. (**B**) The neoplastic cells are weakly positive for LMO2 in a nuclear staining pattern. (**C**-**D**) Higher power view

IHC detection showed that all the classic ACCs displayed a remarkable strong and diffuse cytoplasmic expression of LMO2 (Fig. 2, Table 1). Moreover, the cases with high-grade transformation or poor zymogen granules also showed strong cytoplasmic LMO2 staining (Fig. 3, 4, Table 1). The nuclear expression of LMO2 in germinal center B cells with the lesion could be used as a good internal positive control (Fig. 4B, red arrow), while the other types of lymphocytes without LMO2 expression served as negative controls (Fig. 4B, blue arrow). In contrast, the SC cases showed no cytoplasmic expression pattern or just faint nuclear immunostaining of LMO2 (Fig. 5, Table 2). None of other salivary gland tumors expressed cytoplasmic LMO2 (Table S1). The statistical analysis of the cytoplasmic LMO2 expression showed significant differences between ACCs and other tumors of the salivary gland (Table S2).

## Discussion

The classic form of ACC of the salivary gland usually features a serous acinar cell differentiation that morphologically shows a basophilic appearance with numerous cytoplasmic zymogen granules. However, some tumors are composed of a mixture of different neoplastic cells including serous acinar cells, intercalated duct-resembled cells, vacuolated cells and clear cells. The proportion of serous acinar cells is variable, and ACCs comprising predominantly of intercalated duct-resembled cells or vacuolated cells may show microcystic, cystic or follicular patterns. Sometimes the tumor cells of ACC may even display relatively an eosinophilic color mimicking SCs, which may cause challenges in the differential diagnosis [9, 13–16]. ACC and SC are both low-grade salivary gland cancers and possibly differentiate from secretory cells of the salivary gland through different tumorigenic pathways. Currently, detection of the *ETV6* gene rearrangement is identified as a gold standard for the recognition of SC, and aberrant gene changes like *NR4A3* or *MSANTD3* gene rearrangement have been reported to be related with some ACC cases [17–19]. S-100, mammaglobin, lysozyme and pan-TRK are all valuable IHC markers for the diagnosis of SC, but these antibodies seem  not so specific since which can be expressed in some other tumors of salivary gland [11, 20, 21]. IHC markers for ACC are even fewer, among which DOG1 may be the most useful one. However, it is not specific as well owing to its expression in other salivary gland tumors including adenoid cystic carcinoma, epithelial–myoepithelial carcinoma, salivary duct carcinoma, mucoepidermoid carcinoma, and even some SCs [10, 12]. Periodic acid–Schiff (PAS) staining is frequently applied for highlighting the cytoplasmic zymogen granules, but such a method may sometimes be less sensitive in case of zymogen-poor ACCs [22]. In addition, The nuclear NR4A3 immunostaining introduced more lately by Haller et al. has been recently accepted as a novel diagnostic marker for ACCs [18], however, when we conducted the current study, such an antibody was not commercially available yet. In this study, we discovered cytoplasmic LMO2 staining was intensively positive in ACC cases. Compared with NR4A3, the advantages of LMO2 lie that it has been widely used, especially for the diagnosis of B-cell lymphomas and may be more easily commercially available [7]. In addition, normal expression of this marker in adjacent reactive tissues including serous acinar cells or follicular germinal center-derived B cells may serve as excellent internal positive controls.

LMO2 has been found to be expressed in many normal human tissues and various tumors, and currently, it seems to be a promising marker for identifying germinal center differentiation in B-cell lymphomas [7], distinguishing different types of lymphomas [23], and evaluating the response to treatment [24]. In this study we have discovered for the first time that LMO2 is expressed with high-level in all ACCs, including high-grade transformed and zymogen-poor cases, with an intense cytoplasmic pattern, whereas other commonly seen salivary tumors seem to lack LMO2 expression, which may aid in the distinction between ACC and SC. However the possible biologic correlates of LMO2 overexpression in the tumorigenesis of ACC remains largely unknown, which awaiting further investigations.

Our findings suggest that LMO2 can be used as a novel marker for the recognition of ACC of salivary gland, which may aid in distinguishing ACC from its mimics, especially SC.

## Supplementary Information

**Additional file 1: Supplemetal Table 1.** Summary of the pathological features of tumors in the salivary gland.

**Additional file 2: Supplemental Table 2.** Statistical analysis for cytoplasmic expression of LMO2 between acinic cell carcinomas and other tumors of the salivary gland.

## Data Availability

The datasets generated and/or analyzed during the current study are available from the corresponding author on reasonable request.
