# Supplementary material for: Identification of LMO2 as a new marker for acinic cell carcinoma of salivary gland
Source: Diagn Pathol. 2022 Jan 30;17:15. doi: 10.1186/s13000-022-01192-w (PMC8802521; doi:10.1186/s13000-022-01192-w)
Supplement: Supplementary file 1 — Additional file 1: Supplemetal Table 1. Summary of the pathological features of tumors in the salivary gland. [file 13000_2022_1192_MOESM1_ESM.pdf]

Supplemental Table 1. Summary of the pathological features of tumors in the salivary gland.

Diagnosis	LMO2		DOG1	S-100	*ETV6* rearrangement
	expression	cytoplasmic expression			
Acinic Cell Carcinoma	30/30, C	30/30	29/30	4/30	0/27
Secretory Carcinoma	5/11, N	0/11	3/11	10/11	11/11
Mucoepidermoid Carcinoma	7/22, C, W	7/22, W	8/22	7/22	/
Adenoid Cystic Carcinoma	2/26, C, W	2/26, W	9/26	23/26	/
Polymorphous Adenocarcinoma	2/13, C, W	2/13, W	3/13	12/13	/
Salivary Duct Carcinoma	0/15	0/15	4/15	3/15	/
Basal Cell Adenocarcinoma	0/1	0/1	0/1	0/1	/
Myoepithelial Carcinoma	0/2	0/2	0/2	2/2	/
Epithelial-Myoepithelial Carcinoma	0/2	0/2	0/2	2/2	/
Pleomorphic Adenoma	5/15, N	0/15	4/15	14/15	/
Basal Cell Adenoma	0/15	0/15	10/15	15/15	/
Myoepithelioma	0/1	0/1	0/1	1/1	/
Warthin Tumor	0/15	0/15	0/15	0/15	/
Oncocytoma	0/8	0/8	0/8	0/8	/

C, cytoplasmic; N, nuclear; W, weak
