# Supplementary material for: Identification of LMO2 as a new marker for acinic cell carcinoma of salivary gland
Source: Diagn Pathol. 2022 Jan 30;17:15. doi: 10.1186/s13000-022-01192-w (PMC8802521; doi:10.1186/s13000-022-01192-w)
Supplement: Supplementary file 2 — Additional file 2: Supplemental Table 2. Statistical analysis for cytoplasmic expression of LMO2 between acinic cell carcinomas and other tumors of the salivary gland. [file 13000_2022_1192_MOESM2_ESM.pdf]

Supplemental Table 2. Statistical analysis for cytoplasmic expression of LMO2 between acinic cell carcinomas and other tumors of the salivary gland.

Diagnosis	*P* value
Secretory Carcinoma	<0.001
Mucoepidermoid Carcinoma	<0.001
Adenoid Cystic Carcinoma	<0.001
Polymorphous Adenocarcinoma	<0.001
Salivary Duct Carcinoma	<0.001
Basal Cell Adenocarcinoma	<0.001
Myoepithelial Carcinoma	<0.001
Epithelial-Myoepithelial Carcinoma	<0.001
Pleomorphic Adenoma	<0.001
Basal Cell Adenoma	<0.001
Myoepithelioma	<0.001
Warthin Tumor	<0.001
Oncocytoma	<0.001
